# Enhanced Activation in Phosphorous-Doped Silicon via Dual-Beam Laser Annealing

**DOI:** 10.3390/ma17174316

**Published:** 2024-08-30

**Authors:** Rasheed Ayinde Taiwo, Yeongil Son, Joonghan Shin, Yusuff Adeyemi Salawu

**Affiliations:** 1 Department of Future Convergence Engineering, Kongju National University, 1223-24 Cheonandaero, Seobuk-gu, Cheonan 31080, Republic of Korea; taiwoolore@gmail.com (R.A.T.); yil1554@naver.com (Y.S.); 2 Department of Mechanical and Automotive Engineering, Kongju National University, 1223-24 Cheonandaero, Seobuk-gu, Cheonan 31080, Republic of Korea; 3 Global Institute of Manufacturing Technology (GITECH), Kongju National University, 1223-24 Cheonandaero, Seobuk-gu, Cheonan 31080, Republic of Korea; 4 Department of Physics, College of Natural Science, Daegu University, Gyeongsan 38453, Republic of Korea; salauyusuf25@gmail.com

**Keywords:** dual-laser annealing, phosphorous-doped Si, dopant activation, electrical property, crystalline structure

## Abstract

In this study, we conduct a comparative analysis of single-beam laser annealing (SBLA) and dual-beam laser annealing (DBLA) techniques for semiconductor manufacturing. In the DBLA approach, two laser beams were precisely aligned to simultaneously heat a phosphorus-doped silicon (Si) wafer. The main objective was to investigate the impact of the two annealing techniques on the electrical properties, crystalline structure, and diffusion profile of the treated phosphorus-doped Si at equivalent laser powers. Both SBLA and DBLA improved the electrical properties of the phosphorus-doped Si, evidenced by increased carrier concentration and reduced carrier mobility. Additionally, the crystalline structure of the phosphorus-doped Si showed favorable modifications, with no defects and improved crystallinity. While both SBLA and DBLA produced similar phosphorus profiles with no significant redistribution of dopants compared to the as-implanted sample, DBLA achieved a higher activation ratio than SBLA. Although the results suggest improved dopant activation with minimal diffusion, further studies are needed to clearly confirm the effect of DBLA on dopant activation and diffusion.

## 1. Introduction

The semiconductor industry continually strives for innovations that redefine the boundaries between device performance and integration density [1,2,3]. Silicon (Si) remains an essential material for semiconductor fabrication due to its abundance, excellent electrical properties, and compatibility with existing manufacturing processes [4,5]. A crucial aspect of semiconductor device fabrication is the precise control over the electrical properties of Si through doping, achieved by the intentional introduction of impurities into the Si crystal lattice [6,7]. Among the various doping strategies, the phosphorous (P) doping of Si involves introducing donor impurities such as phosphorous into the Si lattice, a key process in semiconductor fabrication [8,9,10]. The introduction of these donor impurities leads to an increase in the concentration of negative charge carriers by creating additional electrons within the crystal lattice. Phosphorus-doped Si is essential for forming p-n junctions, the cornerstones of diodes and transistors, and for realizing complementary metal–oxide–semiconductor (CMOS) technology, crucial for modern integrated circuits [11,12,13,14,15].

However, precise control over the doping concentration and activation within the Si lattice remains a significant challenge in semiconductor fabrication. Conventional bulk-heating processes, such as furnace annealing or rapid thermal annealing with halogen lamps, often result in significant thermal load and unwanted dopant diffusion. This could greatly reduce the performance of ultra-scaled semiconductor devices (sub-10 nm), where maintaining sharp dopant profiles is crucial for achieving desired electrical characteristics. These limitations are particularly pronounced in cutting-edge semiconductor applications [16,17,18]. In response, laser annealing provides a controlled and localized method to activate dopants without causing extensive damage to the surrounding material, as seen with conventional methods [19,20,21]. One such technique involves using a single-beam continuous-wave (CW) laser [22]. Although single-beam laser annealing (SBLA) offers improved spatial control and a reduced thermal budget compared to conventional methods, it still encounters limitations in achieving precise dopant activation for scaled devices due to the inherent trade-off between achieving high dopant activation levels and avoiding excessive diffusion into the semiconductor device [23,24,25].

A new approach has been proposed in the field of semiconductor device fabrication with the introduction of dual-beam laser annealing (DBLA) for dopant activation [26]. This approach presents a significant opportunity to simultaneously enhance dopant activation efficiency compared to conventional and SBLA methods, while minimizing unwanted dopant diffusion. Such diffusion can significantly impact device performance by causing short-channel effects and increasing leakage currents.

In contrast to SBLA, DBLA employs a combination of CW and pulsed lasers applied to the Si wafers. This dual-beam technique provides a method for enhancing the material properties critical to semiconductor device performance [27]. This unique approach aims to overcome the limitations of both conventional and SBLA techniques, offering enhanced dopant activation with minimal thermal budget. However, it also presents challenges, including the complexity of setup, the precise alignment of two laser beams, and increased costs and maintenance.

Through the strategic application of DBLA, nickel silicide contact was also successfully formed on Si wafer in a microsecond regime [28]. Its applications extend beyond Si and dopant activation, potentially impacting other material properties relevant to semiconductor devices. Although initial studies on DBLA commenced in early 2010, research has been limited, and a comprehensive understanding of the efficacy of DBLA across various materials, dopants, and device architectures remains to be established.

In this paper, we investigate the efficacy of DBLA by employing a combination of CW and pulsed lasers that interact to activate phosphorous dopants in Si. This simultaneous heating treatment enhanced the electrical performance of phosphorus-doped Si by improving dopant activation levels and minimizing undesired dopant diffusion within the crystalline structure. An experiment using the SBLA technique was also conducted for comparative analysis. This comprehensive exploration aims to elucidate the principles, effects, and potential applications of DBLA in phosphorus-doped Si.

The outcomes of our study not only highlight the potential superiority of DBLA in enhancing electrical properties, but also reveal its capability to minimize unwanted dopant diffusion. This advancement could significantly improve the performance of semiconductor devices and reduce leakage currents, marking significant strides in semiconductor technology.

## 2. Experiment

### Materials and Annealing Experiment

A 200 mm wafer of prime-grade, single-side polished p-type Si (100) was used as the base material for the experiment. The wafer was doped with boron with a resistivity of 1–10 Ω·cm, and was obtained via the Czochralski method. Phosphorous ions were implanted onto the substrate surface using a high-current ion implanter (Varian/Applied Materials, VIISta 80HP, Santa Clara, CA, USA) at a 7° tilt angle to minimize channeling effects. The implantation was conducted at a dose of 5 × 10^15^ atoms/cm^2^ and an energy level of 30 keV, resulting in the introduction of phosphorous dopants into the substrate Si (sub-Si) layer. Following ion implantation, the dopant-implanted substrate was precision-cut into 2 × 2 cm sections using a laser cutter. The prepared samples were then subjected to annealing, employing SBLA with a CW fiber laser source (Raycus, RFL-P100MX, Wuhan, China) at a wavelength of 1080 nm, and DBLA using both the CW fiber laser and a pulsed laser (Raycus, RFL-C-2000, Wuhan, China) at 1064 nm. During DBLA, the CW and pulsed laser beams were superimposed on each other, with the pulsed laser beam fixed at a 32° angle to the z-axis. The alignment between the two beams was achieved using a micrometer screw gauge integrated into the pulsed laser setup, ensuring precise and stable positioning of the pulsed laser beam throughout the experiment. Although no real-time monitoring was employed, the setup allowed for accurate initial alignment, ensuring consistent energy delivery during the annealing process.

**Figure 1:**
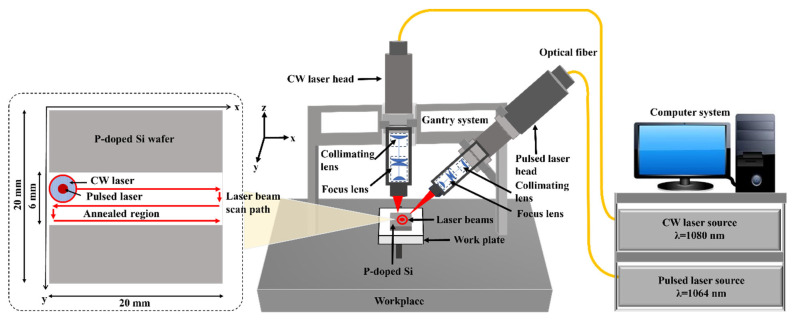
Schematic for DBLA set up. Two laser beams (CW and pulsed laser beam) were inscribed into each other and radiated simultaneously onto phosphorus-doped Si wafer at varied laser power.

As depicted in Figure 1, the two laser beams, originating from the CW and pulsed laser sources, were transmitted via optical fibers to the collimation lenses and subsequently directed onto the sample surfaces through focal lenses. The CW laser head was equipped with collimation and focusing lenses with focal lengths of 100 mm and 200 mm, respectively, whereas the pulsed laser head had collimation and focusing lenses with focal lengths of 100 mm and 250 mm. To achieve the required heating effect, the pulse duration and pulse repetition rate of the pulsed laser were set to 100 ns and 2000 kHz, respectively. Precise control of the laser beam position on the sample surface was achieved utilizing a three-axis motion control system integrated into the laser system.

During annealing, the sample surface was scanned in two directions. The surface was scanned at a speed of 70 mm/s along the primary scan direction (x-direction). The scanning speed along the y-direction, which was used to alter the scan paths, was maintained at 50 mm/s. The CW laser produced a defocused beam with a diameter of 637 μm on the workpiece surface. The separation between the centers of adjacent laser spots on the two consecutive scan paths was kept constant at 250 μm, resulting in a 60% beam overlap of the CW laser beam spots. Additionally, in the DBLA setup, the defocused beam from the pulsed laser, which had a diameter of 94 μm, was combined with the CW laser beam. The pulsed laser spots were aligned such that each spot overlapped with the previous one by approximately 40%.

Table 1 and Table 2 summarize the comparative process conditions for both SBLA and DBLA. The SBLA configuration utilized a laser beam with a power ranging from 220 to 340 W and a scan speed of 70 mm/s. Similarly, the DBLA setup featured laser beams operating within the same total power range of 220–340 W, along with a scan speed of 70 mm/s. Throughout the experiments, the pulsed laser in the DBLA configuration was maintained at a constant power of 20 W. Considering the scan area and scanning speed selected in this study, the process time to scan 6 × 20 mm dimensions was approximately 6.97 s, leading to a rate of 0.058 s/mm^2^. Based on this, the time to scan the whole area of the 300 mm wafer was estimated to be 4106.9 s.

## 3. Sample Analysis Methods

Following laser annealing, the phosphorus-doped Si samples underwent multiple characterizations to evaluate the effects of the annealing process on their electrical, chemical, and microstructural characteristics. The sheet resistances of all specimens were evaluated utilizing a four-point probe method with a CMT-100 M tool. The probe tips were systematically positioned at 1 mm intervals to ensure consistent measurements. For electrical characterization, Hall effect measurements were conducted by subjecting the samples to a magnetic field (B) spanning from −9 to 9 T. The voltage–current (V-I) curve was recorded utilizing a cryogen-free magnet system (Cryogenic Inc., London, UK) at zero magnetic field, with contact electrodes (Cu wires) affixed to the sample surface using conductive silver glue. The microstructural analysis involved transmission electron microscopy (TEM). The samples were prepared using a focused ion-beam (FIB) milling method with a LYRA3 GMH tool (TESCAN, Brno, Czech Republic) at 30 kV. Post FIB milling, the microstructural features of both as-implanted and laser-annealed samples were examined using bright-field-emission TEM with a JEM-F200 microscope (JEOL, Akishima, Japan) operated at 200 kV. Additionally, the distribution and concentration of phosphorous in Si were determined using secondary ion mass spectrometry with an IMS 7f system (Cameca and Ametek, Berwyn, PA, USA). This system enabled the accurate assessment of the phosphorous distribution within the samples. By comparing these characterization results, we analyzed the effects of different annealing conditions on the dopant behavior in both the SBLA and DBLA setups.

## 4. Results and Discussion

### 4.1. Analysis of Sheet Resistance

To analyze the successful dopant activation of implanted phosphorous ions within the near-surface region, we compared the sheet resistance of the as-implanted sample (255 kΩ/sq) with that of the annealed samples for the two annealing techniques. For SBLA, the resistance dropped sharply from 255 kΩ/sq to 11 kΩ/sq at 220 W, indicating successful activation. Further decreases were observed at 240 W (5.7 kΩ/sq), 260 W (2.1 kΩ/sq), 280 W (1.9 kΩ/sq), and 300 W (1.7 kΩ/sq). At 320 W, the resistance decreased to 797 Ω/sq, suggesting ongoing activation. At 340 W, a significant drop to 43.4 ± 0.3 Ω/sq occurred, possibly indicating that the Si layer, amorphized by ion implantation, was recrystallized with improved crystallinity.

Similarly, in the DBLA process, the initial resistance of 10.6 kΩ/sq at a laser power of 220 W indicated dopant activation compared to the resistance of the as-implanted sample (255 kΩ/sq). The resistance decreased continuously, reflecting an increase in dopant activation as the laser power increased. The minimum resistance measured for the DBLA process was 43.4 ± 0.3 Ω/sq at a laser power of 340 W.

**Figure 2:**
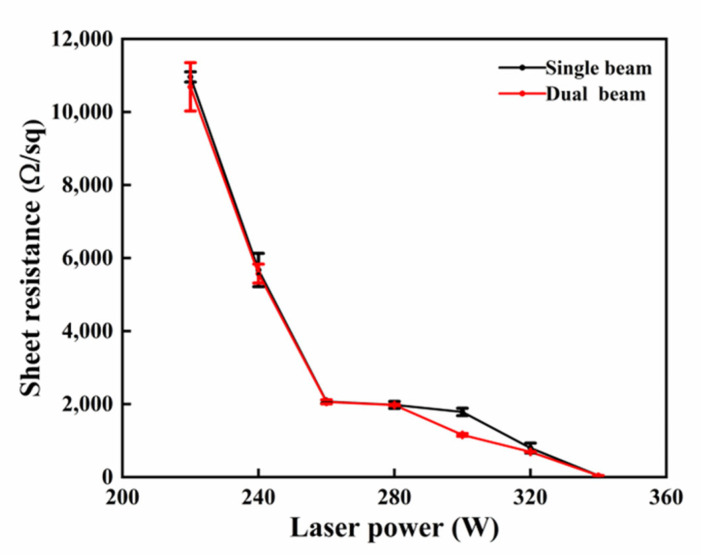
Comparison of sheet resistance measured during SBLA and DBLA. Measurements were taken at different points along vertical and horizontal directions on fabricated sample. Mean value and standard deviation for each condition are presented in graph above.

Comparing the effects of DBLA on resistance reduction with those of SBLA, despite exhibiting similar reduction patterns, DBLA consistently showed marginally lower resistance values than SBLA (Figure 2). This minor variation in resistance values between DBLA and SBLA might indicate subtle differences in the effectiveness of the annealing processes in modifying material properties.

### 4.2. Analysis of Hall Effect Measurements

**Figure 3:**
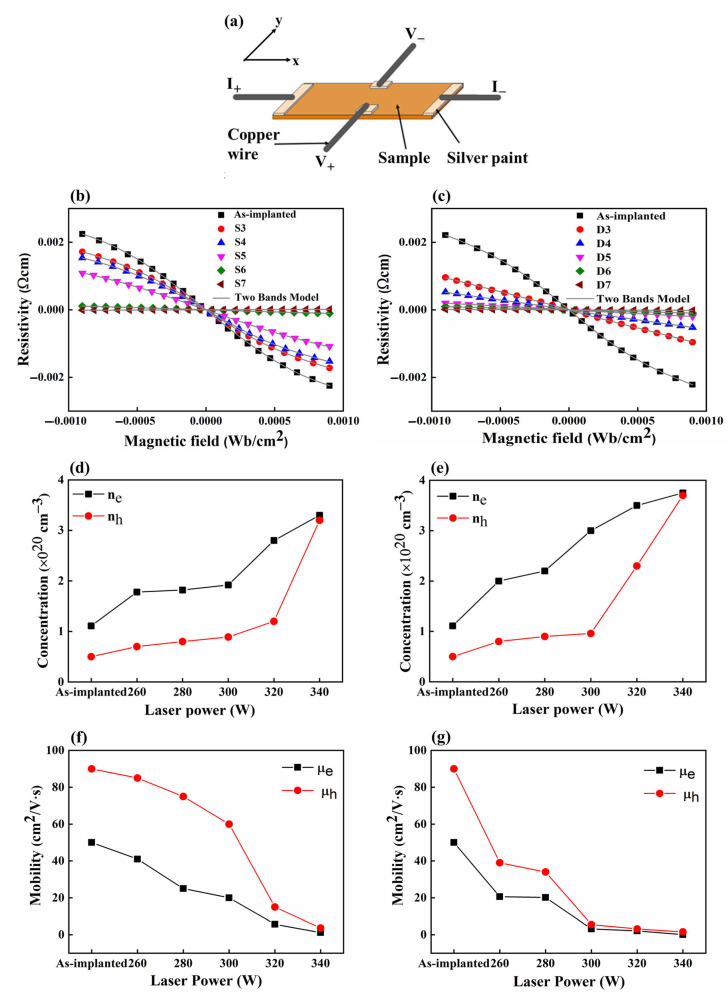
Non-linear Hall effect in phosphorus-doped Si as a function of magnetic fields at various annealed powers: (**a**) schematic for Hall resistance ${\;R}_{xy}$ measurements of as-implanted and annealing samples for both SBLA and DBLA; (**b**), (**d**,**f**) are the evolution of Hall resistivity ${\;\rho }_{xy}$, carrier concentration, and mobility of as-implanted and annealed samples as functions of varied laser power for SBLA, respectively; (**c**), (**e**,**g**) are the evolution of Hall resistivity $\rho_{xy}$, carrier concentration, and mobility of as-implanted and annealed samples as functions of varied laser power for DBLA, respectively.

To gain a deeper insight into the electrical properties of our samples, we employed Hall effect measurements, as shown in Figure 3a. This technique enabled us to determine the carrier concentrations and mobilities of the fabricated materials. In Hall effect analysis, resistivity (*ρ_xy_*) is calculated using the following formula:

(1) $$\rho_{xy}=\frac{VW}{IL}$$

where *V* represents voltage, *L* is the length of the sample in the x-direction, *I* is the current, and *W* is the distance between the voltage probes in the y-direction. Notably, Figure 3b,c show the non-linear characteristics of the *ρ_xy_*(*B*) curves in the magnetic field. This observation challenges the Drude model, which assumes a single carrier type and a linear *ρ_xy_*(*B*) curve. This non-linearity suggests the coexistence of multiple charge carriers. For example, in phosphorus-doped Si, group V elements such as phosphorus introduce electrons as primary negative charge carriers for electrical conduction. Nonetheless, the material also retains intrinsic holes that, once energized, can participate in conduction, thus supporting the inference of multiple-carrier dynamics. According to previous reports [29,30], non-linear *ρ_xy_*(*B*) curves can be quantitatively analyzed using the two-band theory, which is described by the following expression:

(2) $$\rho_{xy}\left(B\right)=\frac{{(n}_{h}\mu_{h}^{2}-n_{e}\mu_{e}^{2})B+{(n}_{h}-n_{e}){\mu_{e}}^{2}{\mu_{h}}^{2}B^{3}}{e{{[(n}_{e}\mu_{e}+n_{h}\mu_{h})}^{2}+{{(n}_{h}-n_{e})}^{2}{\mu_{e}}^{2}{\mu_{h}}^{2}B^{2}]}$$

where *n_h_*(*n_e_*) is the hole (electron) carrier concentration, *μ_h_*(*μ_e_*) is hole (electron) mobility, and e is the elementary charge. As depicted in Figure 3b,c, the *ρ_xy_*(*B*) curves for both as-implanted and annealed samples were well fitted using the two-band model. The strong correlation between these fitted curves and the experimental data suggests that the two-band theory effectively accounts for the non-linear behavior of *ρ_xy_*(*B*) curves. Carrier concentrations and mobilities were quantified at different laser powers, as shown in Figure 3d–g. The results obtained for *n_h_*(*n_e_*) and *μ_h_*(*μ_e_*) from both the SBLA and DBLA techniques exhibit a similar trend. As laser power increased, carrier concentrations also increased, likely due to enhanced dopant activation, while mobilities decreased. This decrease in mobility can be attributed to increased energy delivery to the sub-Si layer during annealing, which promotes impurity scattering due to the enhanced generation of charge carriers within the crystal lattice. In both the SBLA and DBLA techniques, *n_e_* consistently exceeded *n_h_*, signifying more effective donor impurity activation. Although *n_h_* was lower, it also rises with rising laser power, suggesting an enhanced activation of acceptor impurities. At 340 W, *n_e_* and *n_h_* were nearly identical, indicating a saturation point in their activation. Notably, the values of *n_h_*(*n_e_*) obtained from DBLA were higher than those from SBLA, as shown in Figure 3d,e. This difference is attributed to the unique impurity activation mechanism offered by DBLA, where the CW laser mainly acts as a preheating source, elevating the temperature to a level not reached by SBLA alone. The pulsed laser in the DBLA approach, generating laser pulses with high peak power (500 W) and short pulse width (20 ns), even though the average power was only 20 W, produced a high peak temperature in conjunction with the CW laser heating within a short time (20 ns), while minimizing the thermal budget. This high peak temperature likely contributed to higher values of *n_h_*(*n_e_*) compared to SBLA. The differences in mobilities between *μ_e_* and *μ_h_* observed in SBLA were larger than those in DBLA, as shown in Figure 3f,g. This disparity can be explained by the distinct annealing mechanisms each technique employs. In DBLA, the introduction of high peak temperatures by the pulsed laser may ionize previously inactive impurities, creating additional scattering centers for both electrons and holes, which likely contributes to the small difference between *μ_e_* and *μ_h_* as compared to SBLA.

As reported in the literature [31,32], the activated dopant ratio was calculated by dividing the sheet concentration (cm^−2^) by the utilized dose (5 × 10^15^ cm^−2^). To estimate the sheet concentration, a sheet thickness of 113 nm was assumed, consistent with the junction depth discussed in the analysis of the dopant concentration profile (see Section 4.4). Increasing the laser power led to an increase in both the sheet and bulk concentrations, thereby contributing to a higher percentage of activated dopants in both processes, as shown in Table 3. This observation suggests that higher laser power promotes more efficient dopant activation [33,34]. The comparative data also show that DBLA consistently induced a greater activation ratio than SBLA at equivalent power levels. As indicated by Table 3, a 5–24.4% higher activation ratio was obtained by DBLA. This difference highlights the greater efficiency of the dual-beam approach in dopant activation. The enhanced efficiency of the activation ratio can be attributed to the unique combination of CW and pulsed lasers used in DBLA. In the DBLA process, the CW laser with a larger beam spot preheats the surface of sub-Si along the scanning direction. This preheating increases the population of free electrons in the sub-Si and makes the material more receptive to the energy delivered by the following pulsed laser beam. Due to this sequential process, DBLA can cause a higher temperature than SBLA under equivalent laser power, resulting in greater dopant activation. In addition to this, as mentioned previously, the high peak power of the pulsed laser beam likely contributes to the higher temperature and activation of the dopant.

Table 4 shows a comprehensive summary of the Hall effect measurements and the sheet resistance results for both the SBLA- and DBLA-processed samples. The data presented in the table allow for a clear comparison of the key electrical properties obtained by the different laser conditions and annealing methods.

### 4.3. Analysis of Microstructure

**Figure 4:**
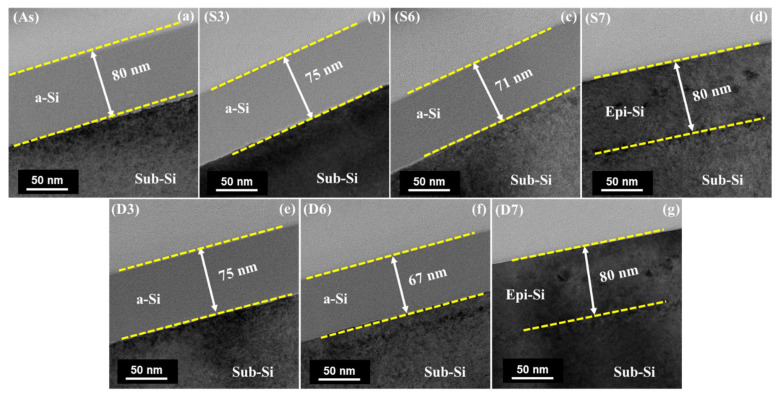
Cross-sectional TEM images of as-implanted and annealed samples: (**a**) as-implanted sample; (**b**–**d**) SBLA samples; (**e**–**g**) DBLA samples.

The TEM cross-sectional images of the as-implanted and annealed samples are shown in Figure 4, illustrating the effects of ion implantation and subsequent laser annealing. The amorphized layer resulting from the ion implantation had a thickness of approximately 80 nm, as shown in Figure 4a. As the laser power increased, a noticeable enhancement in the crystallization process was observed, depicting a clear relationship between laser power and material transformation. Both annealing methods successfully achieved complete crystallization of the entire a-Si layer (80 nm), demonstrating their capability to fully restore the crystalline structure under sufficient laser power. This confirms the effectiveness of laser annealing in inducing recrystallization without visible crystal defects.

The TEM images in Figure 4b–d reveal structural modifications under SBLA. At a laser power of 280 W, as shown in Figure 4b, the thickness of the a-Si layer decreased from 80 to 75 nm. This reduction suggests that the annealing process initiates crystal nucleation and growth, leading to the partial recrystallization of the a-Si layer. However, the extent of recrystallization was limited, as evidenced by the remaining amorphous portion. The a-Si layer remaining in the upper region indicates that recrystallization occurred at the interface between the amorphized layer and the sub-Si. As the laser power increased to 320 W (Figure 4c), the thickness of the a-Si layer further decreased to 71 nm. This indicates a more pronounced recrystallization process, with a greater proportion of the amorphous layer transforming into crystalline Si. At the highest laser power of 340 W (Figure 4d), the entire a-Si layer completely recrystallized. This suggests that the energy delivered by the laser at this power level was sufficient to induce the full recrystallization of the amorphous layer, resulting in the formation of a continuous crystalline Si layer.

The crystal growth observed in the DBLA process was similar to that of SBLA. However, at 320 W (Figure 4f), the DBLA sample shows noticeably enhanced epitaxial growth compared with the SBLA sample at the same power setting. This enhanced growth is attributed to the synergistic effects of the combined laser beams in the DBLA setup. The high peak temperature induced by the pulsed laser in DBLA may enhance the activation of the dopants and facilitate the formation of crystal nuclei, leading to accelerated epitaxial growth.

**Figure 5:**
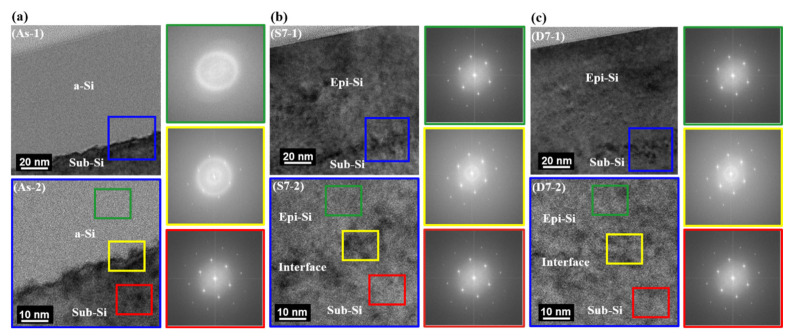
TEM and SAED images of a-Si, Epi-Si, and sub-Si of as-implanted and annealed samples: (**a**) TEM and SAED images for as-implanted sample; (**b**) TEM and SAED images for S7 sample; (**c**) TEM and SAED images for D7 sample (large blue boxes in (**a**–**c**) show enlarged images of areas highlighted by small blue boxes, and green, yellow, and red boxes present SAED areas with corresponding SAED patterns).

The analysis of selected-area electron diffraction (SAED) patterns in specific areas provides crucial insights into the amorphous, interfacial, and monocrystalline layers produced in both the SBLA and DBLA processes, enhancing the understanding derived from TEM analysis. Figure 5 illustrates the electron diffraction patterns of the selected regions.

Figure 5 shows the TEM images and the results of the SAED analysis of the as-implanted Si layer. The large blue box presents three distinct areas within the sample: amorphous silicon (a-Si), the interface, and sub-Si. The diffraction pattern obtained from the a-Si region (identified within the green box) displays a diffuse pattern, characteristic of an amorphous phase, signifying an absence of a crystalline structure and a lack of long-range order. The yellow box indicates an indistinct ring, suggesting a partially amorphized and crystallized region. The red box displays a clear diffraction pattern with bright dots, indicating a single-crystal structure in the Si <011> zone axis, serving as a reference for understanding the diffraction patterns observed in the annealed samples.

Figure 5b illustrates the SAED patterns obtained during SBLA. The area represented by the blue box shows the SAED region with diffraction patterns. The region indicated by the green box shows a well-arranged crystal structure with sharp dots, indicating the recrystallization of Si into a single crystal. The yellow area displays a well-arranged crystal with a diffused ring, suggesting epitaxial growth across the interface between the upper a-Si layer and the lower sub-Si layer. This transition zone is crucial for understanding the interface dynamics during laser annealing. The red area presents a crystal structure like that of sub-Si, confirming epitaxial growth beyond the interface layer. Figure 5c shows similar characteristics for each layer during the DBLA process, mirroring the observations in the SBLA process.

### 4.4. Analysis of Dopant Concentration Profiles

**Figure 6:**
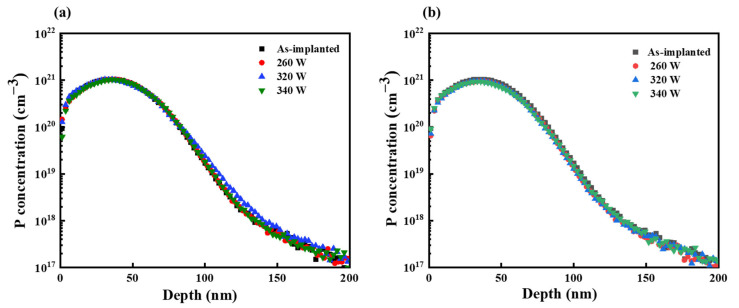
Phosphorous concentration profile along depth direction of Si. As-implanted sample serves as a reference for each condition of the annealing processes: (**a**) SBLA; (**b**) DBLA.

The dopant concentration profiles obtained from the SBLA and DBLA processes revealed nearly identical phosphorous profiles, as shown in Figure 6. The trend of decreasing phosphorous concentrations with depth remained consistent across both techniques. As noted in our previous study [35], junction depth is generally defined as the distance from the surface to the point where the dopant concentration decreases to 5 × 10^18^ cm^−3^. According to this criterion, the junction depth of the annealed samples was 113 nm. Additionally, there was no significant redistribution of the dopants compared to the as-implanted sample. The similarity in the phosphorous profiles suggests that both the SBLA and DBLA processes minimally impact the spatial distribution of phosphorous dopants in the substrate.

Despite this similarity in phosphorous profiles, DBLA demonstrated a higher activation ratio than SBLA. As previously discussed, this difference can be attributed to the synergistic effects of the combined laser beams in DBLA. The simultaneous application of both laser types in DBLA creates a unique thermal environment characterized by steady heating from the CW laser and rapid localized heating from the pulsed laser. This combination of thermal energies enhances dopant activation efficiency, resulting in a higher activation ratio than that achieved with SBLA. This more efficient incorporation of the dopant into the Si lattice using DBLA techniques leads to improved electrical properties of the fabricated material.

## 5. Conclusions

This study reports a comparative investigation of SBLA and DBLA techniques at equivalent laser powers. Both SBLA and DBLA effectively activated the phosphorous dopants in Si, as evidenced by the significant reduction in sheet resistance and the improved crystallinity observed in TEM analysis. Increasing the laser power considerably changed the electrical properties and crystallinity of the annealed samples. However, the overall difference in the effects of the two annealing techniques on the property changes in the material was not significant. Nevertheless, the DBLA process demonstrated a higher activation ratio than SBLA across various laser power settings, while neither annealing method induced a redistribution of phosphorus. This may suggest that DBLA could offer benefits in enhancing the electrical properties of doped Si while minimizing diffusion. As a future extension of this study, simulation will be conducted to better understand the subtle differences between the two processes and to support the analysis of the results presented in this study.

## Figures and Tables

**Table 1:** Process conditions for SBLA.

Experiment No.	CW Laser Power (W)	Scan Speed (mm/s)
S1	220	70
S2	240	70
S3	260	70
S4	280	70
S5	300	70
S6	320	70
S7	340	70

**Table 2:** Process conditions for DBLA.

Experiment No.	CW Laser Power (W)	Pulsed Laser Average Power (W)	Total Power (W)	Scan Speed (mm/s)
D1	200	20	220	70
D2	220	20	240	70
D3	240	20	260	70
D4	260	20	280	70
D5	280	20	300	70
D6	300	20	320	70
D7	320	20	340	70

**Table 3:** Ratios of activated dopant in SBLA and DBLA.

Experiment No.	Laser Power (W)	$\mathrm{Sheet}\;\mathrm{Concentration}\;({\mathrm{cm}}^{-2})$	$\mathrm{Bulk}\;\mathrm{Concentration}\;({\mathrm{cm}}^{-3})$	Activated Ratio (%)
S3	260	2.01 × $10^{15}$	1.78 × $10^{20}$	40.2
S4	280	2.06 × $10^{15}$	1.82 × $10^{20}$	41.2
S5	300	2.17 × $10^{15}$	1.92 × $10^{20}$	43.4
S6	320	3.16 × $10^{15}$	2.80 × $10^{20}$	63.2
S7	340	3.73 × $10^{15}$	3.30 × $10^{20}$	75.0
D3	260	2.26 × $10^{15}$	2.00 × $10^{20}$	45.2
D4	280	2.48 × $10^{15}$	2.20 × $10^{20}$	49.6
D5	300	3.39 × $10^{15}$	3.00 × $10^{20}$	67.8
D6	320	3.95 × $10^{15}$	3.50 × $10^{20}$	79.0
D7	340	4.24 × $10^{15}$	3.75 × $10^{20}$	85.0

**Table 4:** Electrical properties of phosphorous-doped Si measured in this study.

Experiment No.	Laser Power (W)	Resistivity at (0.0009 Wb/cm^2^) $(\times 10^{-4}$ Ωcm)	Sheet Resistance (Ω/sq)	Hole Carrier Concentration $(\times 10^{20}$${cm}^{-3}$)	Electron Carrier Concentration $\left(\times 10^{20}{cm}^{-3}\right)$	Hole Carrier Mobility (cm^2^/V∙s)	Electron CarrierMobility (cm^2^/V∙s)
S3	260	−17.20	2072.50	0.78	1.78	85.00	41.00
S4	280	−15.30	1999.67	0.80	1.82	75.00	25.00
S5	300	−10.80	1786.83	0.89	1.92	60.00	20.00
S6	320	−1.07	797.90	1.20	2.80	15.00	5.70
S7	340	0.14	43.40	3.20	3.30	3.70	1.20
D3	260	−10.00	2050.00	0.80	2.00	39.00	21.00
D4	280	−5.25	1964.17	0.90	2.20	34.00	20.16
D5	300	−2.00	1158.00	0.96	3.00	5.50	3.15
D6	320	−1.00	691.90	2.30	3.50	3.20	2.50
D7	340	−0.14	43.42	3.70	3.75	1.60	0.30

## Data Availability

Datasets generated during the current study are available from the corresponding author on reasonable request.

## References

[1] Radamson H.H., Zhu H., Wu Z., He X., Lin H., Liu J., Xiang J., Kong Z., Xiong W., Li J. (2020). State of the Art and Future Perspectives in Advanced CMOS Technology. Nanomaterials.

[2] Hsieh T.-Y., Hsieh P.-Y., Yang C.-C., Shen C.-H., Shieh J.-M., Yeh W.-K., Wu M.-C. (2020). Single-Grain Gate-All-Around Si Nanowire FET Using Low-Thermal-Budget Processes for Monolithic Three-Dimensional Integrated Circuits. Micromachines.

[3] Son K., Cho K., Kim S., Park S., Jung D.H., Park J., Park G., Kim S., Shin T., Kim Y. (2020). Signal Integrity Design and Analysis of 3-D X-point Memory Considering Crosstalk and IR Drop for Higher Performance Computing. IEEE Trans. Compon. Packaging Manuf. Technol..

[4] Ando T., Fu X.A. (2019). Materials: Silicon and Beyond. Sens. Actuator A Phys..

[5] Ovanesyan R.A., Filatova E.A., Elliott S.D., Hausmann D.M., Smith D.C., Agarwal S. (2019). Atomic Layer Deposition of Silicon-Based Dielectrics for Semiconductor Manufacturing: Current Status and Future Outlook. J. Vac. Sci. Technol. A.

[6] Ryu H.Y., Lee M., Park H., Ko D.H. (2020). Chemical Bonding States and Dopant Redistribution of Heavily Phosphorus-Doped Epitaxial Silicon Films: Effects of Millisecond Laser Annealing and Doping Concentration. Appl. Surf. Sci..

[7] Lee M., Ryu H.Y., Ko E., Ko D.H. (2019). Effects of Phosphorus Doping and Postgrowth Laser Annealing on The Structural, Electrical, and Chemical Properties of Phosphorus-Doped Silicon Films. ACS Appl. Electron. Mater..

[8] Chery N., Zhang M., Monflier R., Mallet N., Seine G., Paillard V., Poumirol J.M., Larrieu G., Royet A.S., Kerdilès S. (2022). Study of Recrystallization and Activation Processes in Thin and Highly Doped Silicon-in-Insulator Layers by Nanosecond Laser Thermal Annealing. J. Appl. Phys..

[9] Zhan X., Su Y., Fu Y., Chen J., Xu H. (2019). Phosphorous-Doped a-Si Film Crystallization using Heat-Assisted Femtosecond Laser Annealing. IEEE Trans. Semicond. Manuf..

[10] Chang R.D., Lin C.H. (2016). Activation and Deactivation of Phosphorus in Silicon-on-Insulator Substrates. Mater. Sci. Semicond. Process..

[11] Gluschenkov O., Liu Z., Niimi H., Mochizuki S., Fronheiser J., Miao X., Li J., Demarest J., Zhang C., Niu C. FinFET Performance with Si: P and Ge: Group-III-Metal Metastable Contact Trench Alloys. Proceedings of the 2016 IEEE International Electron Devices Meeting (IEDM).

[12] Rosseel E., Dhayalan S.K., Hikavyy A.Y., Loo R., Profijt H.B., Kohen D., Kubicek S., Chiarella T., Yu H., Horiguchi N. (2016). Selective Epitaxial Growth of High-P Si: P for Source/Drain Formation in Advanced Si nfets. ECS Trans..

[13] Wu H., Gluschenkov O., Tsutsui G., Niu C., Brew K., Durfee C., Prindle C., Kamineni V., Mochizuki S., Lavoie C. Parasitic Resistance Reduction Strategies for Advanced CMOS Finfets Beyond 7nm. Proceedings of the 2018 IEEE International Electron Devices Meeting (IEDM).

[14] Malka D. (2020). A Four Green TM/Red TE Demultiplexer Based on Multi Slot-Waveguide Structures. Materials.

[15] Malka D., Cohen M., Zalevsky Z., Turkiewicz J. Optical Micro-Multi-Racetrack Resonator Filter Based on SOI WaveGuide. Proceedings of the 2014 IEEE 28th Convention of Electrical & Electronics Engineers in Israel (IEEEI).

[16] Cristiano F., Shayesteh M., Duffy R., Huet K., Mazzamuto F., Qiu Y., Quillec M., Henrichsen H.H., Nielsen P.F., Petersen D.H. (2016). Defect Evolution and Dopant Activation in Laser Annealed Si and Ge. Mater. Sci. Semicond. Process..

[17] Prucnal S., Rebohle L., Skorupa W. (2017). Doping by Flash Lamp Annealing. Mater. Sci. Semicond. Process..

[18] Shima A., Hiraiwa A. (2006). Ultra-Shallow Junction Formation by Non-Melt Laser Spike Annealing and its Application to Complementary Metal Oxide Semiconductor Devices in 65-nm Node. Jpn. J. Appl. Phys..

[19] Tabata T., Karim H., Rozé F., Mazzamuto F., Sermage B., Kopalidis P., Roh D. (2021). Dopant Redistribution and Activation in Ga Ion-Implanted High Ge Content SiGe by Explosive Crystallization during UV Nanosecond Pulsed Laser Annealing. ECS J. Solid State Sci. Technol..

[20] Kim J.H., Ji H.M., Nguyen M.C., Nguyen A.H.T., Kim S.W., Baek J.Y., Kim J., Choi R. (2021). Low-Temperature Dopant Activation using Nanosecond Ultra-Violet Laser Annealing for Monolithic 3D Integration. Thin Solid Films.

[21] Lim S.Q., Williams J.S. (2022). Electrical and Optical Doping of Silicon by Pulsed-Laser Melting. Micro.

[22] Wang Y., Chen S., Shen M., Wang X., Zhou S., Hawryluk A., Hebb J., Owen D. Laser Spike Annealing and its Application to Leading-Edge Logic Devices. Proceedings of the 2008 16th IEEE International Conference on Advanced Thermal Processing of Semiconductors.

[23] He Y., Chen Y., Yu G., Hong A., Lu J.P., Liu X., Yu L., Chen Y. Laser Spike Anneal Macro & Micro Non-Uniformity Investigation using Modulated Optical Reflectance and Four-Point-Probe. Proceedings of the 2010 International Workshop on Junction Technology Extended Abstracts.

[24] Taiwo R.A., Shin J.-H., Son Y.-I. (2022). Comprehensive Analysis of Phosphorus-Doped Silicon Annealed by Continuous-Wave Laser Beam at High Scan Speed. Materials.

[25] Huet K., Aubin J., Raynal P.E., Curvers B., Verstraete A., Lespinasse B., Mazzamuto F., Sciuto A., Lombardo S.F., La Magna A. (2020). Pulsed Laser Annealing for Advanced Technology Nodes: Modeling and Calibration. Appl. Surf. Sci..

[26] Shen X., Wang Y., Wang X. Two-Beam Laser Annealing with Improved Temperature Performance. U.S. Patent.

[27] Wang Y., Chen S., Shen M., Wang X., Zhou S., Hebb J., Owen D. Dual Beam Laser Spike Annealing Technology. Proceedings of the 2010 International Workshop on Junction Technology Extended Abstracts.

[28] Mileham J., Le V., Shetty S., Hebb J., Wang Y., Owen D., Binder R., Giedigkeit R., Waidmann S., Richter I. Impact of Dual Beam Laser Spike Annealing Parameters on Nickel Silicide Formation Characteristics. Proceedings of the 2010 18th International Conference on Advanced Thermal Processing of Semiconductors (RTP).

[29] Huang S.M., Yu S.H., Chou M. (2017). Two-Carrier Transport-Induced Extremely Large Magnetoresistance in High Mobility Sb_2_Se_3_. J. Appl. Phys..

[30] Wang Y., Wang L., Liu X., Wu H., Wang P., Yan D., Cheng B., Shi Y., Watanabe K., Taniguchi T. (2019). Direct Evidence for Charge Compensation-Induced Large Magnetoresistance in Thin WTe2. Nano Lett..

[31] Berencén Y., Prucnal S., Liu F., Skorupa I., Hübner R., Rebohle L., Zhou S., Schneider H., Helm M., Skorupa W. (2017). Room-Temperature Short-Wavelength Infrared Si Photodetector. Sci. Rep..

[32] Berencén Y., Prucnal S., Möller W., Hübner R., Rebohle L., Böttger R., Glaser M., Schönherr T., Yuan Y., Wang M. (2018). CMOS-Compatible Controlled Hyperdoping of Silicon Nanowires. Adv. Mater. Interfaces.

[33] Das D., Patra C. (2023). Superior Phosphorous Doping in Nanocrystalline Silicon Thin Films and their Application as Emitter Layers in Silicon Heterojunction Solar Cells. Energy Fuels.

[34] Kennedy N., Duffy R., Eaton L., O’Connell D., Monaghan S., Garvey S., Connolly J., Hatem C., Holmes J.D., Long B. (2018). Phosphorus Monolayer Doping (MLD) of Silicon on Insulator (SOI) Substrates. Beilstein J. Nanotechnol..

[35] Taiwo R.A., Shin J.-H., Son Y.-I. (2023). Evaluating Suitability of Green Laser Annealing in Developing Phosphorous-Doped Silicon for Semiconductor Devices. Mater. Sci. Semicond. Process..

